# Isolation, Identification and Genomic Analysis of Orange-Spotted Grouper Iridovirus Hainan Strain in China

**DOI:** 10.3390/v16101513

**Published:** 2024-09-24

**Authors:** Helong Cao, Dongzhuo Zhang, Guanghui Mu, Siting Wu, Yurong Tu, Qiwei Qin, Jingguang Wei

**Affiliations:** 1 College of Marine Sciences, South China Agricultural University, Guangzhou 510642, China; chl6707@163.com (H.C.); sting23332021@163.com (S.W.); 2 Guangdong Winsun Biological Pharmaceutical Co., Ltd., Guangzhou 511356, China; 18826254943@163.com (D.Z.); muguanghui109@126.com (G.M.); tuyurong29@163.com (Y.T.); 3 Nansha-South China Agricultural University Fishery Research Institute, Guangzhou 511400, China; 4 Laboratory for Marine Biology and Biotechnology, Pilot National Laboratory for Marine Science and Technology (Qingdao), Qingdao 266000, China

**Keywords:** *Epinephelus coioides*, OSGIV-HN-2018-001, megalocytivirus, isolation, characterization

## Abstract

The orange-spotted grouper (*Epinephelus coioides*) is an important mariculture fish in China. However, in recent years, with the rapid development of aquaculture activities, outbreaks of viral diseases have affected the grouper aquaculture industry, causing severe economic losses. In the present study, we isolated and identified a virus from diseased, orange-spotted groupers from an aquaculture farm in Hainan Province, China. The isolated virus was identified as orange-spotted grouper iridovirus, hence named the orange-spotted grouper iridovirus Hainan strain (OSGIV-HN-2018-001). OSGIV-HN-2018-001 induces a cytopathic effect after the infection of mandarin fish (*Siniperca chuatsi*) brain clonal passage (SBC) cells. In addition, the cytoplasm of the OSGIV-HN-2018-001-infected SBC cells was found to contain a large number of hexagonal virus particles with a diameter of approximately 134 nm. Using the Illumina NovaSeq system, we assembled the sequence data and annotated the complete genome of OSGIV-HN-2018-001 (GenBank accession number: PP974677), which consisted of 110,699 bp and contained 122 open reading frames (ORFs). Phylogenetic tree analysis showed that OSGIV-HN-2018-001 was most closely related to ISKNV-ASB-23. The cumulative mortality rate of groupers infected with OSGIV-HN-2018-001 reached 100% on day 8. The spleens were enlarged and blackened after the dissection of the dying groupers. These results contribute to the understanding of the molecular regulatory mechanism of the iridovirus infection and provide a basis for iridovirus prevention.

## 1. Introduction

Grouper aquaculture is one of the most important marine aquaculture industries in China. According to the China Fishery Statistics Yearbook 2024, the production of groupers in 2023 was 241,400 tonnes, accounting for 11.74% of the country’s total marine fish aquaculture production. This was a remarkable increase of 17.33% over the previous year, and the current grouper aquaculture production in China has been increasing steadily over the years. At present, grouper aquaculture in China is mainly concentrated in Guangdong, Hainan, and Fujian provinces, which together account for 96.96% of the total grouper aquaculture production in China [1]. However, for a long time, frequent diseases in grouper farming, especially viral diseases caused by the iridovirus infection, have seriously threatened the health of the fish and the sustainable development of the grouper industry.

Iridoviridae is a family of large, icosahedral viruses with double-stranded DNA (dsDNA) genomes ranging in size from 103 to 220 kbp. The virions have icosahedral symmetry and diameters ranging from 120 to 200 nm [2]. Members of the Alphairidovirinae and Betairidovirinae subfamilies can infect ectothermic vertebrates (bony fish, amphibians, and reptiles), insects, and crustaceans [3]. Based on the viral genome GC content, major capsid protein (MCP) gene similarity, clinical disease, and other key characteristics, the family Iridoviridae is divided into seven genera [4,5,6], namely Iridovirus, Chloriridovirus, Lymphocystivirus, Ranavirus, Megalocytivirus, Decapodiridovirus, and Daphniairidovirus.

Among them, the megalocytivirus is one of the most important viral pathogens of fish, causing high economic losses to the fish farming industry worldwide [7]. It was reported that red seabream iridovirus disease (RSIVD) was the first outbreak of megalocytivirus-induced disease in 1990 [8]. Subsequently, other megalocytiviruses were discovered, such as rock bream iridovirus (RBIV) [9,10], infectious spleen and kidney necrosis virus (ISKNV) [11], and turbot reddish body iridovirus (TRBIV) [2,12]. Based on phylogenetic analysis and serological studies, the genus megalocytivirus can be divided into two species: ISKNV and Scale Drop Disease Virus (SDDV) [13,14]. Furthermore, the ISKNV species includes a large cluster represented by three genotypes: RSIV, ISKNV, and TRBIV. As research has progressed, an increasing number of megalocytiviruses have been discovered, expanding the diversity within the megalocytivirus family to include an orange-spotted grouper iridovirus (OSGIV) [15], a Taiwan grouper iridovirus (TGIV) [16], and a large yellow croaker iridovirus (LYCIV) [17,18]. In addition, an SDDV-close European chub iridovirus (ECIV) and an unclassified three-spined stickleback iridovirus (TSIV) have recently been isolated and classified [19,20,21]. Megalocytiviruses cause systemic infections that can lead to significant losses in many different species of freshwater and marine fish, both in cultured and wild stocks [22]. Therefore, the isolation and identification of megalocytiviruses play a crucial role in the prediction and prevention of viral disease outbreaks.

In this study, a megalocytivirus was isolated and identified from diseased grouper samples collected in Hainan Province, China. We named it the orange-spotted grouper iridovirus Hainan strain (OSGIV-HN-2018-001). The complete genome sequence of OSGIV-HN-2018-001 was then annotated and analyzed, and its pathogenicity was also investigated. This study provides information to further investigate the interaction between the grouper and the virus.

## 2. Materials and Methods

### 2.1. Cells and Fish

Mandarin fish (*Siniperca chuatsi*) brain clonal passage (SBC) cells were constructed from mandarin fish brains and grown at 28 °C in Leibovitz’s L-15 medium (Gibco, Grand Island, NY, USA) supplemented with 6% fetal bovine serum (Gibco). The cells were provided by Dongzhuo Zhang.

Healthy pearl groupers (*Epinephelus lanceolatus*) with an average length of 7.8 ± 0.3 cm and weight of 10.8 ± 0.4 g were obtained from fisheries in Shandong Province, China. They were kept in a recirculating system at 24–28 °C and fed twice daily for one week. Three groupers were then randomly selected to determine whether the fish were infected with bacteria or viruses.

### 2.2. Collection of Sick Fish Samples

Diseased groupers with an average length of 15 cm were obtained from an aquaculture farm in Hainan Province, China. The fish were quickly necropsied, and tissues such as the liver, spleen, and kidney were collected and frozen in liquid nitrogen and stored at −80 °C.

### 2.3. Virus Detection and Isolation

The tissue from the diseased fish were used for DNA extraction. Briefly, genomic DNA was extracted using the TIANamp Genomic DNA Kit (Tiangen Biotech, Beijing, China).

Megalocytivirus major capsid protein (MCP)-specific primers, MCP-F1 (5′-CTAGTGGA-GGAGGTGTCGGTGTC-3′) and MCP-R1 (5′-GCAGGCGTTCCAGAAGTCAAGG-3′), were designed using Primer Premier 5 for PCR analysis [23]. The PCR reaction conditions were as follows: a cycle of 94 °C for 3 min, 30 cycles of 94 °C for 30 s, 56 °C for 30 s, and 72 °C for 1 min, and a final extension step at 72 °C for 10 min. The PCR products were detected by 1% agarose gel electrophoresis.

Spleen tissues from the diseased groupers were homogenized in sterile PBS and filtered through a 0.22 µm filter. The filtrate was freeze-thawed three times and then incubated with SBC cells. Cytopathological effects (CPEs) were observed under a microscope. The infected cells were collected as virus pools. Viral titers were determined using the TCID_50_ assay [24].

### 2.4. Transmission Electron Microscopy Assay

OSGIV-HN-2018-001-infected SBC cells were harvested, fixed with 2.5% glutaraldehyde in 0.1 M phosphate buffer (pH 7.4) overnight at 4 °C, washed twice with 0.1 M phosphate buffer (pH 7.4) and post-fixed with 1% osmium tetroxide. The samples were then dehydrated through a graded ethanol series, embedded in resin, and sectioned. The sections were double stained with uranyl acetate and lead citrate. Grids of ultrathin sections were examined at 120 kV using a Talos L120C transmission electron microscope (Thermo Fisher Scientific, Waltham, MA, USA). Photographs were taken with a CDD camera.

### 2.5. Genome Sequence Determination

The sequencing was performed on the Illumina Novaseq platform. ORFs were predicted using Geneious Prime 2024.0.5 software. According to the protein sequences of the four core-conserved proteins, the phylogenetic tree of the DNA polymerase protein, MCP, ATPase protein, and ribonuclease III protein was constructed using the neighbor-joining method with 1000 bootstrap replicates in MEGA11.0 software. Gene collinearity analysis based on the physical location of the genome and protein similarity was performed with Geneious Prime 2024.0.5 software.

### 2.6. Fish Experimental Infection

Twenty healthy groupers were injected intraperitoneally with 10^6.7^ TCID_50_/mL of virus suspension (infection group). The control group was injected with sterile PBS. The fish were placed in 300 L of artificial freshwater and maintained at 28 °C. The dead fish were recorded daily, and cumulative mortality was calculated based on the data collected up to 10 days post-infection (p.i.).

## 3. Result

### 3.1. Isolation and Identification of Virus

In our investigation of the grouper epidemic in Hainan Province, we found that the symptoms of many groupers were similar to those caused by megalocytivirus. Compared with healthy groupers, the diseased fish showed loss of appetite, hypoxia, and abnormal swimming. After necropsy, there was obvious enlargement of the spleen and kidney tissues, which was very similar to the symptoms after megalocytivirus infection. Accordingly, liver, spleen, and kidney tissues were collected, DNA was extracted, and a PCR reaction was performed with MCP primers from megalocytivirus. The gel electrophoresis results showed a band of 1362 bp, which was the expected size. The PCR products were sent to a biological company for sequencing, and the sequencing results matched the MCP sequence of the megalocytivirus. Therefore, the virus was named the orange-spotted grouper iridovirus Hainan strain (OSGIV-HN-2018-001).

**Figure 1:**
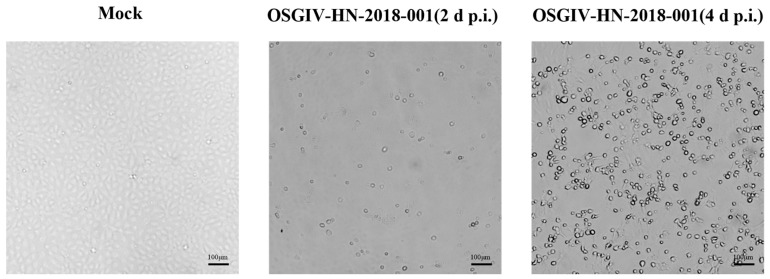
OSGIV-HN-2018-001 infection in SBC cells. CPE induced by OSGIV-HN-018-001 in SBC cells. SBC cells were infected with OSGIV-HN-018-001 at indicated time points (2 d p.i. and 4 d p.i.). Cell morphologies were observed by light microscopy.

**Figure 2:**
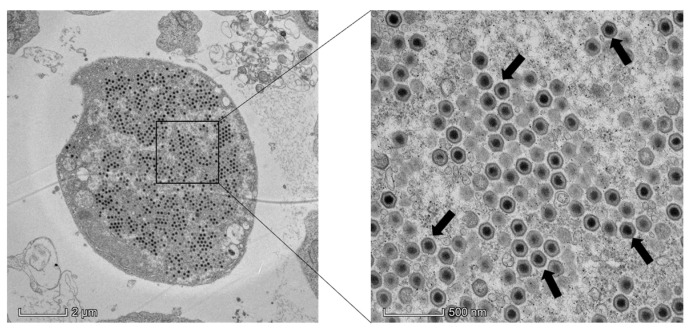
Viral particles were examined by electron microscopy.

In order to better understand the infection mechanism of the virus, the new virus strain was isolated and identified. First, the filtrate of spleen tissue from diseased fish was added to SBC cells in accordance with the sensitivity of SBC cells to megalocytivirus infection. After three generations, some cells were pathological. The infected cells were collected, frozen, and thawed several times as the original solution for the virus infection. The viral solution was then added to the SBC cells at a concentration of 1:1000. As shown in Figure 1, some cells became rounded and swollen. After 4 days, obvious CPE was visible. Electron microscopy revealed numerous hexagonal virus particles (approximately 134 nm diameter) in the cytoplasm of SBCs at day 4 p.i. (Figure 2).

### 3.2. Genetic Characterization

**Figure 3:**
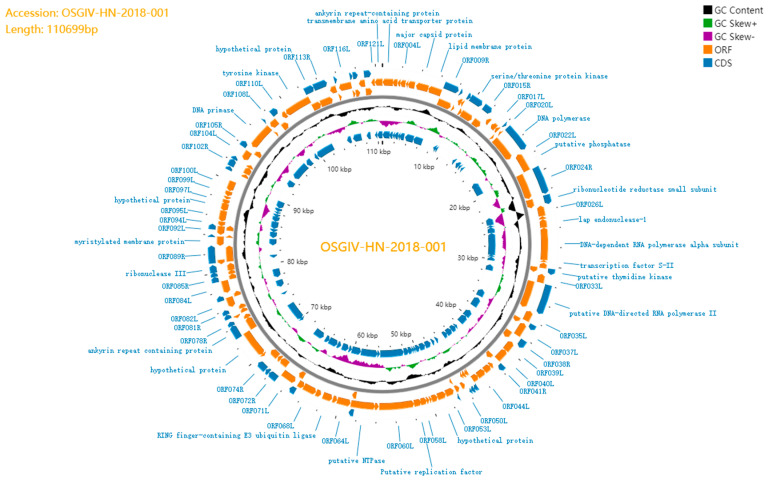
OSGIV-HN-2018-001 genome circle mapping using the online software Proksee. The orange portion represents the predicted open reading frame (ORF), with the arrow direction indicating the approximate size and transcription direction of the ORFs. The blue part is the information on CDs, which were subjected to a homology search using the NCBI BLASTp (https://blast.ncbi.nlm.nih.gov/Blastp (accessed on 20 September 2024)) program. This circle diagram also shows the CG content and CG Skew distribution of the genome.

OSGIV-HN-2018-001 was sequenced on the Illumina Novaseq platform. The full length of OSGIV-HN-2018-001 is 110,699 bp, and its GenBank accession number is PP974677. It has a GC content of 55%, which is in line with the average GC content of a megalocytivirus. The genome of OSGIV-HN-2018-001 has a total of 122 open reading frames, which encode for at least 120 amino acids, of which 26 are core-conserved proteins (Figure 3). There are 47 ORFs in the clockwise direction and 75 ORFs in the counterclockwise direction if the transmembrane amino acid transporter gene is assigned as the first ORF (counterclockwise). Among the 122 ORFs, 105 ORFs had the highest homology with ISKNV-ASB-23, 84 of which had 100% homology; the remaining 17 ORFs had high homology with LBUSV-GZ, BCIV-2015, and SKIV-SD, 6 of which had 100% homology with LBUSV-GZ.

### 3.3. Phylogenetic Tree and Genome Structure of OSGIV-HN-2018-001

**Figure 4:**
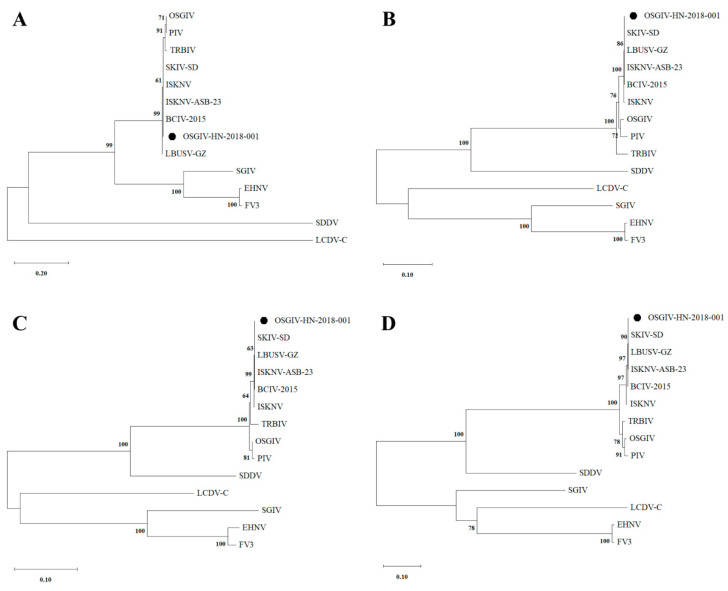
Phylogenetic relationship of OSGIV-HN-2018-001 and other fish iridoviruses. Phylogenetic trees based on the amino acid sequences of DNA polymerase (**A**), MCP (**B**), ATPase (**C**), and ribonuclease III (**D**) were constructed using MEGA software. All virus sequences were taken from the GenBank database (accession number: EHNV, FJ433873; FV3, NC_005946; ISKNV, AF371960; LCDV-C, AY300826; OSGIV, AY894343; PIV, MK098185; SDDV, MN562489; SGIV, AY521625; TRBIV, GQ273492; ISKNV-ASB-23, PP151097; LBUSV-GZ, OP009387; BCIV-2015, MW883595; SKIV-SD, MT986830).

**Figure 5:**
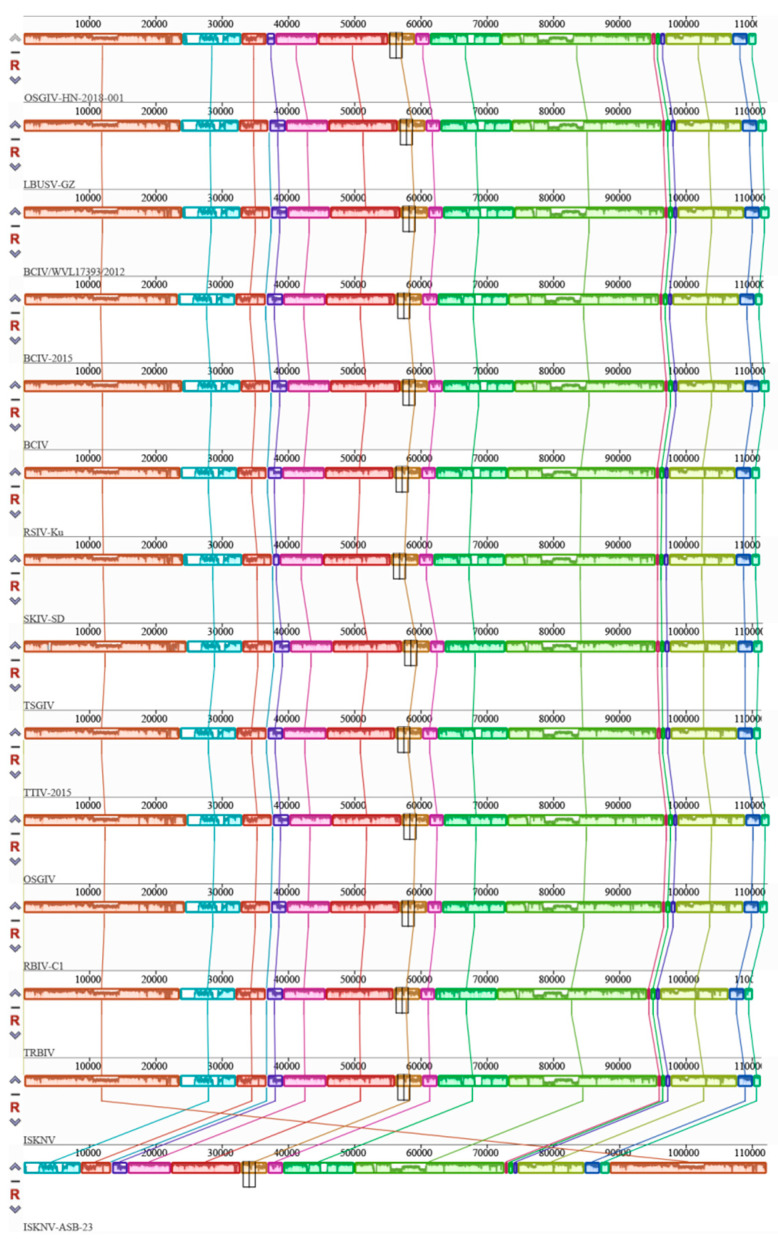
Genome collinearity between OSGIV-HN-2018-001 and other megalocytiviruses. Data were obtained from the Mauve 2.3.1 software that analyzes population evolutionary events through multiple sequence comparisons. Accession numbers of viral genomes are as follows: LBUSV-GZ, OP009387; BCIV/WVL17393/2012, MN432490; BCIV, MN432490; BCIV-2015, MW883595; RSIV-Ku, KT781098; SKIV-SD, MT986830; TSGIV, MG570132; TTIV-2015, MW883599; OSGIV, AY894343; RBIV-C1, KC244182; TRBIV, GQ279492; ISKNV, AF371960; ISKNV-ASB-23, PP151097.

Phylogenetic trees for DNA polymerase, MCP, ATPase, and ribonuclease III were constructed using the MEGA 11.0 software. These trees were constructed from the protein sequences of four conserved core iridovirus proteins. The results indicate that OSGIV-HN-2018-001 is closely related to ISKNV-ASB-23, LBUSV-GZ, and SKIV-SD (Figure 4). In order to better compare and analyze the genome structure of OSGIV-HN-2018-001 and other megalocytiviruses, we performed gene collinearity analysis on the basis of the physical location of the genome and protein similarity. The results show that the physical location of the genome of OSGIV-HN-2018-001 was completely consistent with that of BCIV, LBUSV-GZ, SKIV-SD, and several other genotypes (Figure 5), and it is noteworthy that OSGIV-HN-2018-001 and ISKNV-ASB-23 had the highest homology in ORF prediction, but there was a crossed arrangement of homology patches in the collinearity map, which may be due to different transcription directions and different definitions of the first ORF. In conclusion, OSGIV-HN-2018-001 is an ISKNV-type virus.

### 3.4. Experimental Infection of OSGIV-HN-2018-001 in Grouper

**Figure 6:**
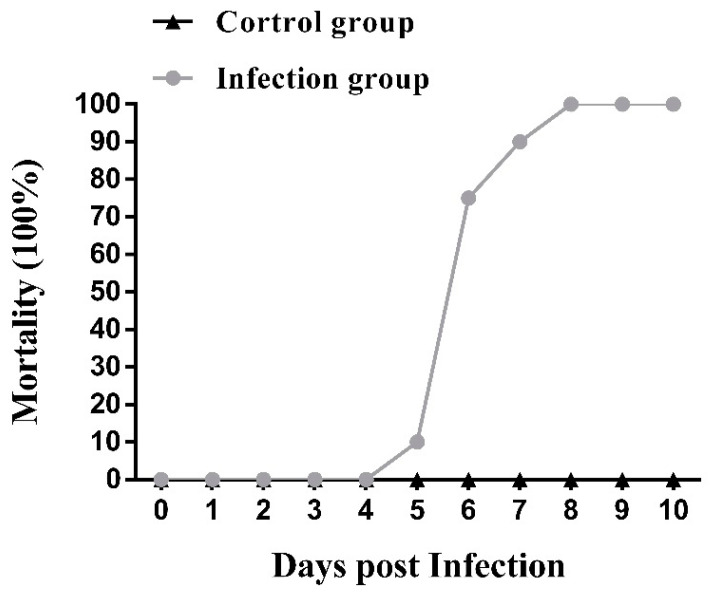
Pathogenicity of OSGIV-HN-2018-001 in groupers. Cumulative mortality of groupers after infection with OSGIV-HN-2018-001 was recorded up to 10 d p.i.

**Figure 7:**
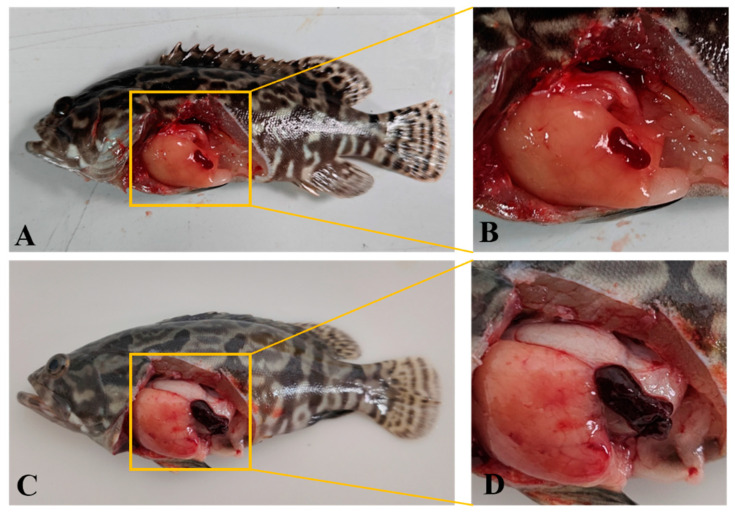
Healthy and clinical signs of groupers infected with OSGIV-HN-2018-001. (**A**,**B**) Healthy groupers; (**C**,**D**) groupers infected with OSGIV-HN-2018-001.

**Figure 8:**
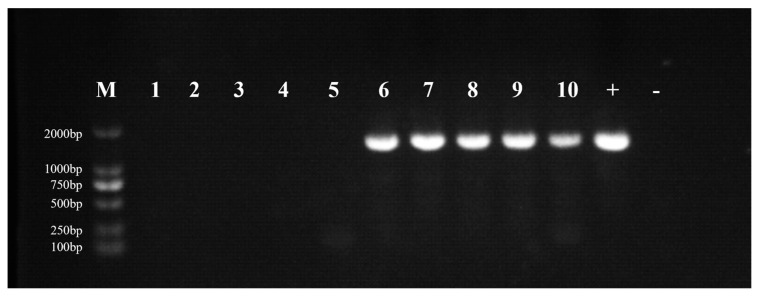
The results of PCR. The spleens of normal and dying groupers were taken for PCR. Agarose (1%) gel showed the PCR product (1362 bp) (lanes 1–5: normal fish in the control group; lanes 6–10: dead fish in the infected group), “+” lane is the positive control and “-” lane is the negative control.

After infection with OSGIV-HN-2018-001, the grouper mortality was recorded daily. Mortality in the infected group started on day 5, peaked on days 6–7, and reached a cumulative mortality rate of 100% on day 8 (Figure 6). The pathological features were the same as those of natural infection. Compared with normal groupers, the diseased groupers showed pathological phenomena such as anorexia, abnormal swimming, and sluggishness, and the spleens were enlarged and blackened after dissection (Figure 7), whereas no groupers died in the control group. The spleens of normal and dying groupers were taken for PCR. The results show that distinct MCP bands were detected in the dying groupers, and the size of the bands was as expected, whereas there were no bands in the control group (Figure 8).

## 4. Discussion

The megalocytivirus is one of the most important viral pathogens of fish. This virus causes severe systemic disease with significant mortality in cultured marine fish for human consumption and freshwater ornamental fish [25]. The megalocytivirus has a wide range of hosts, including fish belonging to 50 different species that live in both freshwater and marine environments [26].

In the present study, it was observed that the spleens of diseased groupers showed hypertrophy. DNA was then extracted from the corresponding tissues and amplified using PCR. The PCR product was then subjected to nucleic acid electrophoresis followed by sequencing analysis, which showed that the size of the band was consistent with that of the megalocytivirus.

Following processes such as grinding, filtration, and additional purification steps, the tissue samples were added to SBC cells for infection. After several generations of blind passage, conspicuous CPE became apparent. The virus’s infection solution was then frozen at −20 °C as the original virus infection solution. Under the electron microscope, a hexagonal megalocytivirus particle with a diameter of approximately 134 nm was observed. According to previous studies, the diameters of some megalocytiviruses are as follows: the viral particles of ISKNV-ZY are hexagonal with a diameter of approximately 131 nm [23]. Under electron microscopy, the viral particles of SKIV-SD are approximately 140 nm in diameter [24]. Megalocytiviruses are large linear single dsDNA genomes with large icosahedral DNA measuring 120–200 nm in diameter [27,28]. Therefore, the electron microscopy results were consistent with the morphological characteristics of the megalocytivirus.

We then sequenced the whole genome of the virus and named it OSGIV-HN-2018-001. The sequencing results show that the total gene length was 110,699 bp, and the GC content was 55%, which is consistent with the average value of megalocytivirus (110–113 kb, ~55%) [4]. According to NCBI comparison results, the virus has the highest homology with ISKNV-ASB-23. A total of 122 ORFs were predicted by the whole gene, of which 84 ORFs had 100% homology with ISKNV-ASB-23, and the rest were related to other megalocytiviruses such as LBUSV-GZ, SKIV-SD, and BCIV-2015, which have high homology.

To date, isolated megalocytiviruses have been classified by the International Committee on Taxonomy of Viruses (ICTV) into 3 genotypes, including genotype 1 (RSIV group), genotype 2 (ISKNV group), and genotype 3 (TRBIV group) [8,29,30]. Importantly, studies have shown that ECIV and SDDV have a longer genome (>128 kb) and lower GC content (<40%) compared to the above three genotypes, suggesting that they may represent a novel fourth genotype of megalocytivirus (genotype 4, SDDV group) [13,31].

Studies have confirmed that the MCP and ATPase genes are commonly used in the phylogenetic analysis of many cytomegalovirus isolates [4]. According to the results of the phylogenetic tree analysis in our study, the megalocytivirus can be divided into two large clusters, ISKNV and SDDV, confirming previous studies [2]. OSGIV-HN-2018-001 is in a branch of ISKNV and is closely related to ISKNV-ASB-23, LBUSV-GZ, SKIV-SD, BCIV-2015, and other megalocytiviruses. Furthermore, the results of the gene collinearity map analysis show that OSGIV-HN-2018-001 is completely consistent with ISKNV-ASB-23, LBUSV-GZ, SKIV-SD, and BCIV-2015 genotypes. These results indicate that OSGIV-HN-2018-001 is indeed a strain of ISKNV.

In order to further investigate the pathogenicity of OSGIV-HN-2018-001, we conducted a virus-infected grouper experiment. The experimental results show that the mortality of the infected groupers started on the 5th post-infection day, with the peak mortality occurring on the 6th and 7th days, and all died on the 8th day. Studies have shown that after megalocytivirus infection of fish and basophil enlargement in multiple organs is a typical manifestation [4]. In this study, control groupers and dead groupers were dissected. It was found that the spleens of the dead groupers were obviously swollen and blackened, and the results of the PCR show that the dead groupers had obvious bands while the control group did not, which also indicates that OSGIV-HN-2018-001 is highly pathogenic.

## Data Availability

The datasets generated for this study are available on request to the corresponding author.
